# Gleaning Information from a “Thank You”: How Witnessing Expressions of Gratitude Shape Perceptions of and Actions Toward Others

**DOI:** 10.1007/s42761-026-00389-2

**Published:** 2026-07-15

**Authors:** Alexandra M. Gray, David DeSteno

**Affiliations:** Department of Psychology, Northeastern University, Boston, MA 02115 USA

**Keywords:** Gratitude, Emotion expression, Halo effect, Strangers, Impression formation

## Abstract

Gratitude expressions of recipients toward benefactors prompt third-party witnesses to ascribe moral qualities to benefactors. To date, however, relatively little is known about how observing the gratitude expressions of others toward their benefactors affects moral perceptions of expressers. Given that gratitude is a moral emotion, its expression should signal an expresser’s moral qualities, and possibly by extension, a halo of positive traits outside the moral domain. Here, we measured how the expression of gratitude impacts inferences about expressers’ moral and general positive traits (i.e., halo effects) and how such perceptions shape observers’ desires to affiliate and cooperative behavior in a trust dilemma with expressers. Across two studies (*N* = 680), participants watched a video depicting a benefactor resolving a problem for an expresser who conveyed either gratitude or simple positivity in return, and then provided impressions of the expresser, and, in the second study, decided whether to cooperate with the expresser to earn money where exploitation by the expresser was possible. We found witnessing gratitude expressions produced stronger moral and nonmoral positive halo effects than witnessing simple positivity expressions and were principally mediated by perceptions of greater responsiveness from expressers. These halos, in turn, increased trust, perceived closeness, and desires to interact with expressers, with trust directly influencing decisions about whether to cooperate on a financial task entailing risk. The findings have implications for the group-level function of gratitude in signaling expresser traits and helping witnesses identify high-quality social partners.

**Supplementary Information:**

The online version contains supplementary material available at https://doi.org/10.1007/s42761-026-00389-2.

People make judgements about an unfamiliar target’s intentions and dispositional traits based on thin slices, or brief observations of their behavior (Ambady & Rosenthal, 1992; Frank, 1988; Jones et al., 2006; Keltner & Haidt, 1999; Knutson, 1996; Reis et al., 1990; Thornton, 1943; Trope, 1986). One such observable behavior is the expression of gratitude in recognition of another person’s kindness or favor (McCullough et al., 2001).

As one might expect, initial work suggests people who witness a gratitude expression believe the expresser possesses some broad qualities that position them as a desirable social partner (Algoe et al., 2020; Fehr et al., 2025; Percival & Pulford, 2020; Ritzenhöfer et al., 2017). Work by Algoe and colleagues (2020) offered initial evidence that third-party witnesses are willing to provide more help to and self-disclose personal information to an unfamiliar gratitude expresser than to someone who expressed sentiments of general positivity or affective neutrality in a relevant situation.

The question, of course, is: Why? Investigations into the mechanism underlying these phenomena have suggested perceived responsiveness of expressers (i.e., the extent to which the expresser appears to understand, validate, and care for the benefactor; Reis et al., 2004) plays an important role in mediating affiliative intentions and behaviors toward those who express gratitude (Algoe et al., 2020). However, we believed an additional unrecognized step in the causal chain exists: trait influences from thin slices of behavior. That is, we suspected an expression of gratitude engenders perceived responsiveness by showcasing the expresser’s other-focused behavior in that moment—their praise and concern for the benefactor—and leads to specific sets of trait inferences of expressers that support their responsive behavior and credible character, which subsequently mediate witnesses’ prosocial and affiliative behaviors toward the grateful person.

This view expands the earlier model put forth by Algoe and colleagues (2020) by incorporating an economic and trait-inference based model proposed by Robert Frank (1988). As Frank posited, expressions of morally-toned emotions signal a person possesses certain traits underlying a stable sense of morality—a sense they could be trusted to limit self-interest and cooperate in trustworthy ways. To date, little evidence exists to show whether witnesses of gratitude expressions ascribe such discretely defined moral traits to expressers (Fehr et al., 2025; Percival & Pulford, 2020; Ritzenhöfer et al., 2017) and whether inferring such traits underpin economically risky prosocial behaviors. According to Unkelbach and colleagues (2008), it is possible moral trait attributions bring to mind nonmoral positive traits. However, earlier work has not explored any gratitude-specific (i.e., as compared to the expression of affective positivity) inferences of positive traits in general (e.g., warmth, competence) nor differentially assessed moral and nonmoral positive trait inferences.

To unite extant models of how and why witnessing gratitude expressions leads to prosocial intentions and actions toward expressers, we conducted two studies building off the framework set forth by Algoe and colleagues (2020). Like them, we expected third-party witnesses of a gratitude expression versus one of general positivity perceive expressers as more responsive to others’ beneficial acts. However, we also assessed distinct sets, or halos, of moral and nonmoral positive traits to ascertain whether gratitude expressions as thin slices of behavior lead, as Frank theorized (1988), to differential trait inferences that subsequently shape affiliative and prosocial behavioral intentions. Finally, in the second study, which served as a replication and extension of the first, we examined if and how such trait inferences guide prosocial behavior in a trust dilemma with financial risk. That is, we sought to determine whether trait inferences stemming from gratitude expressions would actually lead to prosocial behaviors, as opposed to intentions, where a partner could cause financial loss.

## Study 1: Witnessing Gratitude Promotes Affiliative Attitudes and Intentions

The goals of Study 1 were two-fold. First, to establish whether third-party witnesses perceive two trait halo effects, one with moral traits and the other with general positive traits, of gratitude expressers and to determine whether these halos are partially a function of the perceptions of the grateful person’s responsiveness. Second, to assess whether the trait halos mediate witnesses’ affiliative attitudes and intentions toward the gratitude expresser, focusing on trust, perceived closeness, proximity seeking, and willingness to capitalize (i.e., a form of social sharing in which someone discloses a past personal positive event or personal good news) with the expresser.

## Method

### Participants

We had set the target sample size using the smaller of the two effect sizes for the moral halo and expanded positive halo effects found in a pilot study (*d* = 0.276). We believed this estimate was appropriate, as it also reflected what is commonly viewed as a relatively small effect size and thus would result in power to detect most effects of interest. Given this estimate, we initially calculated that a sample of 318 participants was needed to achieve power = 0.80 at an alpha level = 0.05 for comparing means of two independent groups. We then inflated the sample size by 15% to account for any data quality issues (i.e., failure on the attention check by some participants), thereby setting the target sample size to be 366. We subsequently recruited this number of participants from a sample of adults living in the United States (U.S.) using the online recruiting platform Prolific (Prolific, 2026). Participants were required to have a 95%+ approval rating and fluency in English. We excluded 19 participants with incomplete data and five for failing to correctly answer an attention check question that asked participants to identify the nature of the workplace problem that was presented in the video. We next excluded 13 participants for survey completion times that were flagged as extreme (note that this exclusion criterion was not part of the pre-registration, but we applied it in the same manner to both experiments given the clear indication that participants had been attending to other things while completing the study). We identified these extreme outliers following Tukey’s method (Tukey, 1977) of calculating the outer fence boundaries on the upper side of the interquartile range for the target variable of interest, which here was the completion time for the survey. Participants with values beyond the outer fence were identified as extreme outliers and excluded from the analyses, given the probability of their being a normal part of the full distribution was less than 1%.^1^  

The final sample consisted of 335 participants aged 18–85 years (*M* = 43.62, *SD* = 13.56). There was an even split of 165 (49%) males and 161 (48%) females. Three individuals self-described and six did not provide their gender. The ethnic composition of the sample was as follows: 69.55% White/Caucasian, 14.63% Black/African American, 4.18% East Asian, 2.99% Multiracial, 2.69% Southeast Asian, and 1.19% South Asian. Six participants did not provide their race and 10 self-described.

Unfortunately, however, we realized after submitting the pre-registration and collecting all data that our sample size calculations contained an error, and therefore did not provide the expected level of power for the noted effect size. We therefore conducted a sensitivity analysis to determine the minimum effect size for which we would achieve a power equal to 0.80 based on the obtained sample. We are pleased to report that the resulting effect size is *d* = 0.31 – a value only slightly discrepant from the intended one.

## Procedure

Participants completed an online survey with a video depicting the resolution of a workplace problem and several questionnaires. Upon opening the survey, participants were first randomly assigned to either the Positive Affect Expression Condition (*n* = 168) or Gratitude Expression Condition (*n* = 167). The conditions differed by which emotional expression, either positivity or gratitude, the expresser conveyed to the benefactor in the video. Previous work has statistically controlled for perceived positive affect of the expresser when examining perceived responsiveness as a mechanism for witnesses’ intentions to help and affiliate with the expresser (Algoe et al., 2020). That is, the distinction between gratitude and positivity was based on statistically controlling for the positivity of a given gratitude expression. We believed that a better, direct comparison between the two expressions was necessary to determine gratitude’s distinct signal value, as different emotional expressions convey disparate information about the expresser and how they feel about the recipient of the expression. To control for any impression effects that might generally apply to any expression of a positively-valenced emotion, we tested the unique signal value of a gratitude expression. Additionally, we aimed to mirror a real-life scenario where witnesses observe the event evoking gratitude between the benefactor and beneficiary followed by a gratitude expression. Third-party witnesses in the current research watched video stimuli portraying an entire gratitude interaction with fully embodied expressions. The two videos were normed on perceived positivity of the emotional expressions. These analyses are included in the Supplementary Materials.

At the beginning of the survey, text explained that participants would watch the resolution of a workplace problem and rate how the workers deal with the issue. They were then told they had been randomly selected to watch the resolution of a technical problem and, unknown to the workers in the video, the experimenters programmed one of the computers to malfunction, jeopardizing the worker’s progress on their task. In actuality, all participants watch the resolution of the same technical issue, and the workers were confederates (i.e., actors) trained to follow a scripted set of behaviors.

In the two-minute video, two female workers sat side-by-side at different desks with their backs facing the camera and computer screens visible. The worker on the left, labeled as “Worker 1,” was the expresser and the worker on the right, labeled “Worker 2,” was the benefactor. Next, text on the video explained the workers were completing long client review presentations and Worker 1 was nearly finished. Then, Worker 1’s computer appeared to malfunction. Worker 1 tried fixing the issue by clicking computer keys and moving the mouse around. She also looked around at the surrounding desks and sighed in frustration. Worker 2 leaned over and asked what happened. Worker 1 responded claiming the computer crashed and implied they might have to make their presentation again. Worker 2 offered to help, and after checking computer wires, moving the mouse, and pressing different keys, Worker 1’s presentation appeared on the screen again. Worker 1 turned to Worker 2, their expression visible to the camera, and expressed positivity in the Positive Affect Expression Condition, “Wonderful! I was so close to finishing, so this is great!” while gesturing toward Worker 2 with one hand. In the Gratitude Expression Condition, Worker 1 expressed gratitude, “Thank you so much! I was so close to finishing, so I really appreciate your help!” while placing their hand on their chest.

After watching the video, participants completed questionnaires capturing their perceptions of the expresser’s and benefactor’s moral traits (i.e., moral halo) and nonmoral positive traits (i.e., expanded positive halo), the expresser’s level of gratitude, perceived expresser responsiveness, affiliative attitudes and intentions toward the expresser, assumptions about the expresser’s social network and interpersonal relations, and demographic information. At the end of the survey, participants were fully debriefed. The results for examining the impact of both halos on witness assumptions about the expresser’s social network and interactions with others are reported in the Supplementary Materials (see Table S1) as these analyses were not of primary interest.

### Measures

#### Moral Halo

Witness perception of the moral halo effect for the expresser consisted of an aggregate of seven moral traits used previously to measure witness perceptions of benefactor moral goodness (Algoe et al., 2020) with an additional three traits to represent a more comprehensive construct of moral character. The 10 moral traits were considerate, honest, helpful, generous, sincere, fair, dependable, trustworthy, kind, and humble. Participants were asked to rate the extent to which they believed these traits described Worker 1 using a 7-point scale from 0 (*not at all*) to 6 (*extremely*). These ratings were averaged to form the expresser moral halo effect (Cronbach *α* = 0.96). Participants additionally reported their perceptions of a moral halo on the benefactor, but these analyses were not part of the primary analyses of interest and are presented in the Supplementary Materials (see Figure S1).

#### Expanded Positive Halo

Witness perception of the expanded positive halo effect on the expresser was a composite of nine positive traits outside the moral domain, many of which are frequently studied as halo effect traits (e.g., Albright et al., 1988; Batres & Shiramizu, 2022; Fritsch et al., 2023; Langlois et al., 2000). The general positive traits were intelligent, competent, collaborative, optimistic, extraverted, warm, supportive, enthusiastic, and attractive. Participants were asked to rate the extent to which they believed these traits described Worker 1 using a 7-point scale from 0 (*not at all*) to 6 (*extremely*). These perceptions were averaged to form the expresser expanded positive halo effect (Cronbach *α* = 0.94). We also captured witness perceptions of an expanded positive halo on the benefactor, and the results are reported in the Supplementary Materials (see Figure S1).

#### Perceived Expresser Responsiveness

Perceived expresser responsiveness measured witness beliefs about the expresser’s understanding, validation, and care for the benefactor. Participants responded to three items that were modified from a perceived responsiveness scale for new acquaintances (Reis et al., 2011) to fit the present study’s cover story of observing unknown workers. Participants were asked how much they thought Worker 1 “seemed to understand Worker 2,” “expressed liking and encouragement for Worker 2,” and “seemed to value Worker 2’s abilities and opinions.” Responses were made on a 7-point scale from 0 (*not at all*) to 6 (*very true*). Responses to the items were averaged to calculate witness perceived responsiveness of the expresser (Cronbach *α* = 0.88).

#### Trust

Witness trust of the expresser was measured with a single item, “How much do you trust Worker 1?” Participants responded on a 7-point scale from 0 (*not at all*) to 6 (*extremely*).

#### Closeness

Witnesses reported their perceived closeness with the expresser by answering one question asking, “How close do you feel to Worker 1?” Participants rated their closeness on a 7-point scale from 0 (*not at all close*) to 6 (*extremely close*).

#### Proximity Seeking

Motivation to seek proximity, or engage in relationship-promotion behaviors, with the expresser was measured using the proximity seeking scale from Peng and colleagues (2018) with adapted wording to fit the context of observing workers. Participants indicated their level of agreement with three statements on a 7-point scale from 1 (*strongly disagree*) to 7 (*strongly agree*). Each item began with “If given the opportunity” and ended with a statement: “I would like to work on the same team with Worker 1,” “I would like to share a workspace with Worker 1,” and “I would like to get to know Worker 1.” Ratings of the items were averaged to calculate witness proximity seeking with the expresser (Cronbach *α* = 0.96).

#### Willingness to Capitalize

Participant desire to capitalize, or disclose positive events or good news about the self, with the expresser was measured with a single item, “How willing are you to share your own positive events or good news with Worker 1?” Participants responded on a 7-point scale from 0 (*not at all willing*) to 6 (*very willing*).

#### Perceived Gratitude

As a manipulation check, participants were asked “How grateful do you think Worker 1 felt?” and responded on a 7-point scale from 0 (*not at all grateful*) to 6 (*extremely grateful*).

## Results

We report descriptive statistics of the variables used in this study. Means, standard deviations, and correlations of these variables are presented in Table 1.

**Table 1:** Means, Standard Deviations, and Correlations of Study 1 Variables

Variable	*M*	*SD*	1	2	3	4	5	6	7
1. Expression Type	0.49	0.50							
2. Moral Halo	3.97	1.23	0.22^***^						
3. Expanded Positive Halo	3.78	1.13	0.18^**^	0.83^***^					
4. Perceived Responsiveness	4.51	1.31	0.23^***^	0.65^***^	0.58^***^				
5. Trust	3.99	1.24	0.19^***^	0.70^***^	0.67^***^	0.60^***^			
6. Closeness	1.77	1.48	0.09	0.49^***^	0.48^***^	0.36^***^	0.45^***^		
7. Proximity Seeking	4.79	1.35	0.19^***^	0.75^***^	0.71^***^	0.60^***^	0.73^***^	0.54^***^	
8. Willingness to Capitalize	3.69	1.51	0.16^**^	0.61^***^	0.58^***^	0.58^***^	0.61^***^	0.56^***^	0.70^***^

*M* and *SD* are used to represent mean and standard deviation, respectively

^***^p< .01, ^**^p < .01, ^*^p< .05

### Manipulation Check

To ensure participants believed the gratitude expression indicated more gratitude than the positivity expression, we compared the perceived level of gratitude conveyed by the expresser across expression types using an independent samples t-test. The manipulation of expressions was successful. Witnesses rated the expresser in the gratitude video as expressing more gratitude (*M* = 5.48, *SD* = 0.90) than the expresser in the positivity video (*M* = 4.82, *SD* = 1.44), *t*(331) = 4.99, *p* < .001, *d* = 0.55, 95% CI [0.33, 0.77].

### Halo Effects as a Function of Emotional Expression

**Fig. 1:**
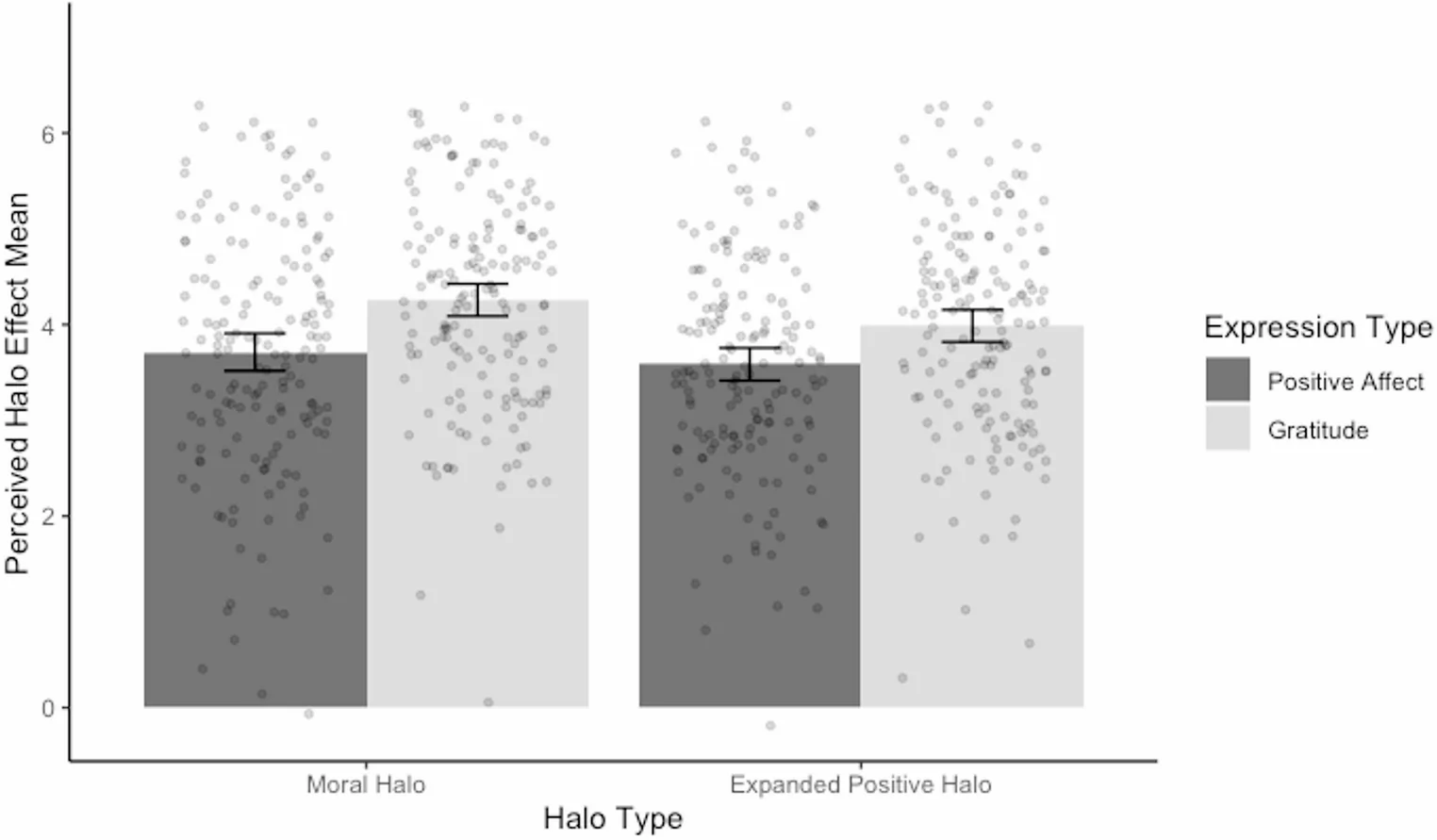
Stronger Moral and Expanded Positive Halo Effects for the Gratitude Expresser. *Note*. Witness perceptions of the moral halo and expanded positive halo of the expresser were significantly higher when observing a gratitude expression compared to a positivity expression (*p* < .01). ^*a*^ Error bars represent 95% confidence intervals

We first ran a 2 × 2 mixed ANOVA to evaluate the effect of expression type (gratitude vs. positivity) and halo type (moral halo vs. expanded positive halo) on third-party witnesses’ perceptions of halo effects on the expresser. The results indicated a significant main effect for expression type, *F*(1, 333) = 15.43, *p* < .001, ηp^2^ = 0.04; significant main effect for halo type, *F*(1, 333) = 27.49, *p* < .001, ηp^2^ = 0.08; and no significant interaction between expression type and halo type, *F*(1, 333) = 3.561, *p* = .06, ηp^2^ < 0.01 (see Fig. 1). Next, we performed separate independent samples t-tests to assess whether the moral halo and expanded positive halo effects differed between witnessing a gratitude expression and positivity expression. Witnesses perceived a stronger moral halo for the worker who expressed gratitude (*M* = 4.26, *SD* = 1.10) than for the worker who simply expressed positivity (*M* = 3.71, *SD* = 1.29), *t*(333) = 4.16, *p* < .001, *d* = 0.45, 95% CI [0.24, 0.67]. Witnesses also perceived a stronger expanded positive halo for the gratitude expresser (*M* = 3.99, *SD* = 1.10) than for the positivity expresser (*M* = 3.59, *SD* = 1.12), *t*(333) = 3.31, *p* = .001, *d* = 0.36, 95% CI [0.15, 0.58].

## Perceived Expresser Responsiveness as Mediator of Halo Effects

**Fig. 2:**
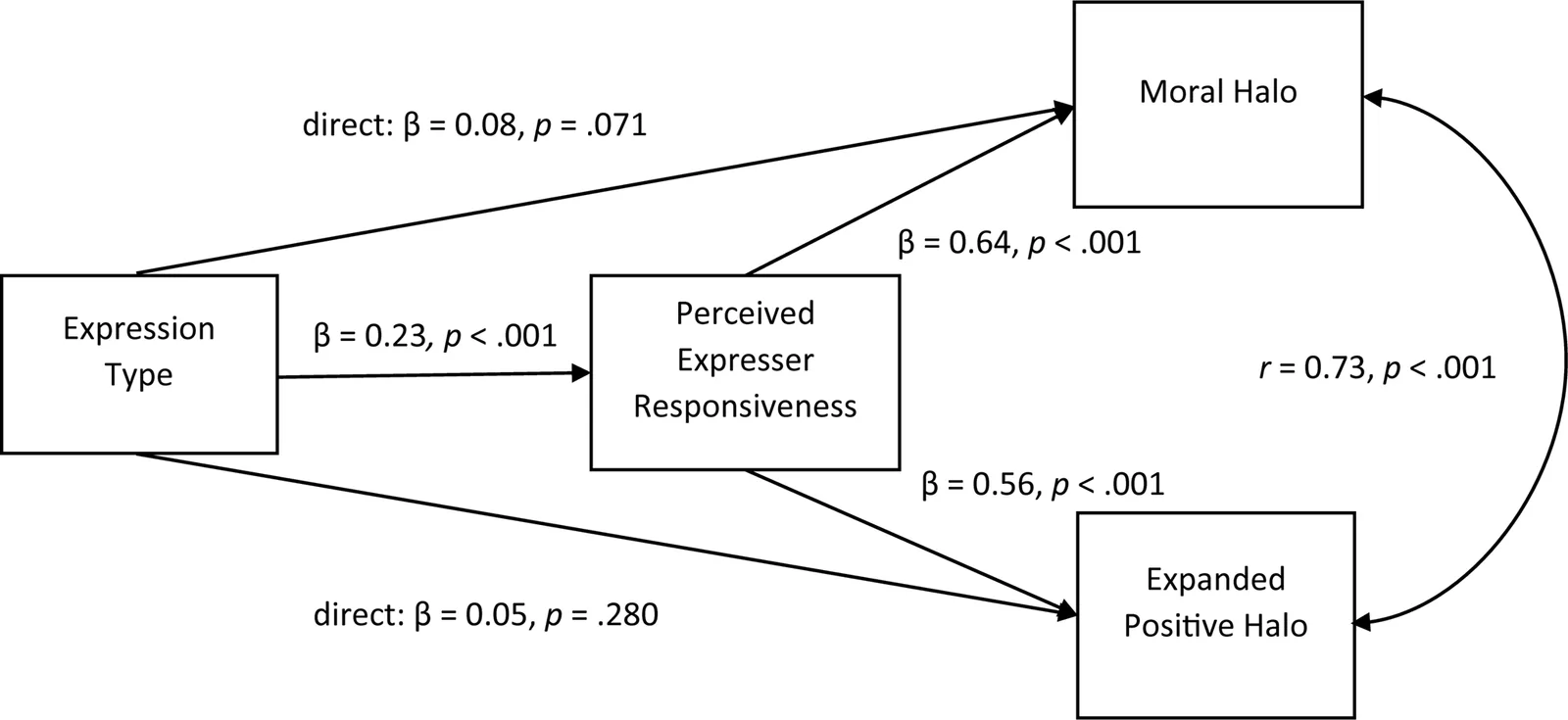
Perceived Expresser Responsiveness Mediates Expression Type and Halo Effects for the Gratitude Expresser. *Note*. Perceived expresser responsiveness mediated the effect of observing a gratitude expression on witness perceptions of the moral and expanded positive halo effects for a gratitude expresser. ^*a*^Expression type was coded as 0 = Positive Affect Expression and 1 = Gratitude Expression

To determine if the perceived responsiveness of expressers mediated the effect of emotion expression type on the halos, we estimated parameters for the path model depicted in Fig. 2. Here, the type of emotion expression did influence perceived responsiveness, such that witnesses believed the gratitude expresser to be more responsive than the positivity expresser. In turn, perceived responsiveness influenced both halos in a positive manner.^2^ Suggesting complete mediation, expression type had no direct influence on either halo outside of its indirect effect through perceived responsiveness (moral halo indirect effect: *b* = 0.36, *SE* = 0.09, *p* < .001, β *=* 0.15, 95% CI [0.08, 0.21]; expanded positive halo indirect effect: *b* = 0.29, *SE* = 0.07, *p* < .001, β *=* 0.13, 95% CI [0.07, 0.19]; total effect: *b* = 0.95, *SE* = 0.24, *p* < .001, β *=* 0.40, 95% CI [0.21, 0.59]). Due to a fully saturated model, RMSEA = 0.

## Halos as Mediators of Affiliative Attitudes and Intentions Toward the Expresser

**Fig. 3:**
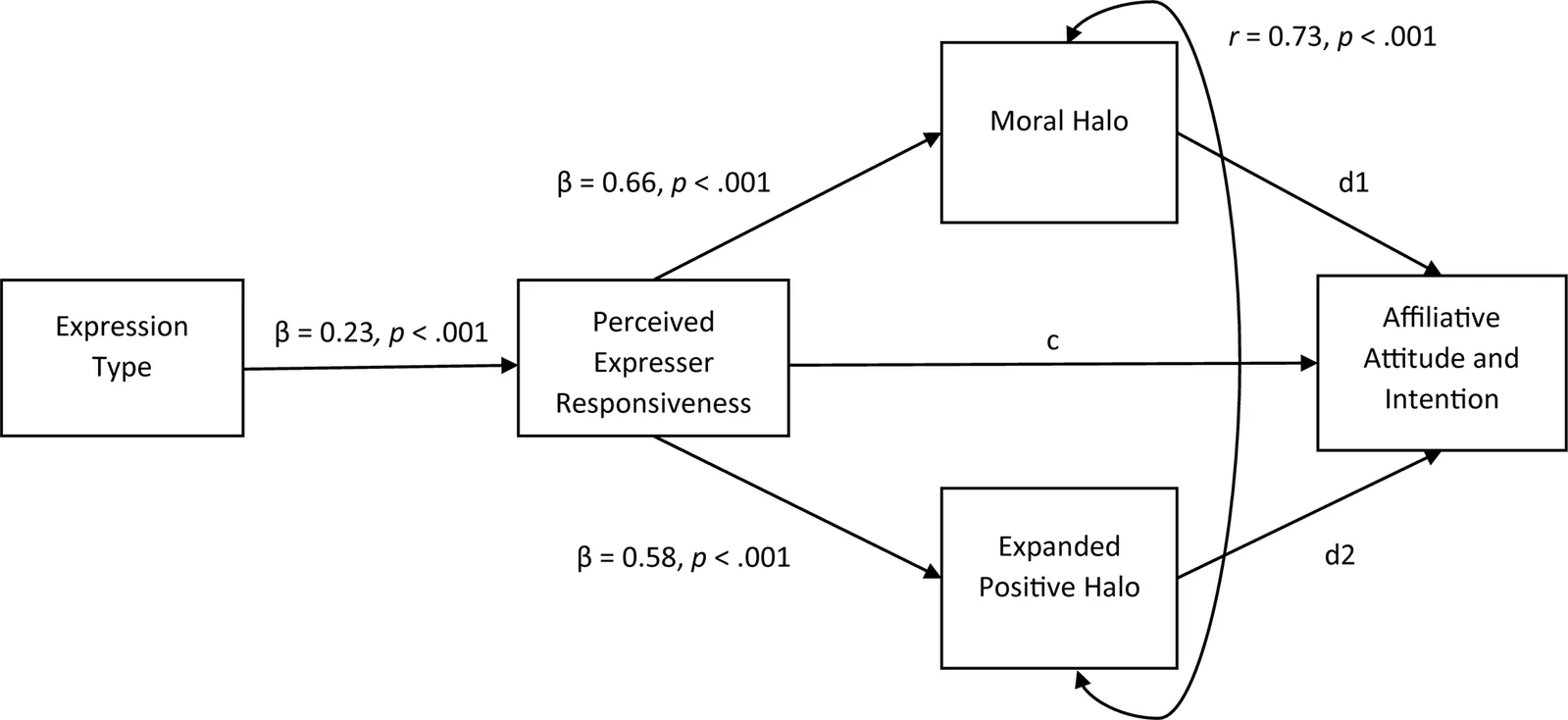
Perceived Expresser Responsiveness and Subsequent Halo Effects Mediate Expression Type and Affiliative Attitudes and Intentions Toward the Gratitude Expresser. *Note*. Witnesses perceived greater responsiveness from a gratitude expresser than a positivity expresser, which, in turn, promoted moral and expanded positive halo effects for the gratitude expresser, further motivating affiliative attitudes and intentions toward the gratitude expresser. ^*a*^Expression type was coded as 0 = Positive Affect Expression and 1 = Gratitude Expression. ^*b*^This is a simplified model with the variances of the halo effects correlated. The double-headed curved arrow represents this correlation

To ascertain the impact that each halo exerts uniquely on perceptions relevant for intentions to engage with expressers, we estimated a series of path models of the form depicted in Fig. 3, where the parameters c, d1, and d2 refer to the specific parameters for a given affiliative attitude and intention variable as noted in Table 2. In the previous analysis, the direct paths from expression type to both halo effects were not significant, indicating full mediation of expression type on both halo effects through perceived responsiveness. Consistent with this finding and to maintain parsimony, these direct paths have been removed in all subsequent mediation models.

As with the previous path analysis, expression type predicted greater perceived expresser responsiveness (*b* = 0.60, *SE* = 0.14, *ß* = 0.23, *p* < .001, 95% CI [0.13, 0.33]) and perceived expresser responsiveness predicted a greater moral halo (*b* = 0.61, *SE* = 0.04, *ß* = 0.66, *p* < .001, 95% CI [0.59, 0.72]) and greater expanded positive halo (*b* = 0.49, *SE* = 0.04, *ß* = 0.58, *p* < .001, 95% CI [0.50, 0.65]). In all cases, path analyses revealed complete or partial mediation of the link between expression type and each affiliative attitude and intention through perceived responsiveness and, simultaneously, the halo effects. Expression type demonstrated significant indirect effects through perceived responsiveness and both the moral halo (indirect effect: *b* = 0.11, *SE* = 0.04, *p* = .003, β *=* 0.05, 95% CI [0.02, 0.08]) and expanded positive halo (indirect effect: *b* = 0.09, *SE* = 0.03, *p* = .003, β *=* 0.04, 95% CI [0.01, 0.06]) on trust (RMSEA = 0.02, PCLOSE = .629, 90% CI [0, 0.10]). Similarly, expression type showed significant indirect effects through perceived responsiveness and the moral halo (indirect effect: *b* = 0.11, *SE* = 0.05, *p* = .023, β *=* 0.04, 95% CI [0.01, 0.07]) and expanded positive halo (indirect effect: *b* = 0.09, *SE* = 0.04, *p* = .021, β *=* 0.03, 95% CI [0.01, 0.06]) on perceived closeness (RMSEA = 0.03, PCLOSE = .611, 90% CI [0, 0.10]). Expression type yielded significant indirect effects through perceived responsiveness and the moral halo (indirect effect: *b* = 0.17, *SE* = 0.05, *p* = .001, β *=* 0.06, 95% CI [0.03, 0.10]) and expanded positive halo (indirect effect: *b* = 0.09, *SE* = 0.03, *p* = .005, β *=* 0.04, 95% CI [0.01, 0.06]) on proximity seeking (RMSEA = 0.02, PCLOSE = .628, 90% CI [0, 0,10]). Lastly, we observed significant indirect effects for expression type on willingness to capitalize with the expresser through perceived responsiveness and the moral halo (indirect effect: *b* = 0.10, *SE* = 0.04, *p* = .017, β *=* 0.04, 95% CI [0.01, 0.06]) and expanded positive halo (indirect effect: *b* = 0.08, *SE* = 0.04, *p* = .020, β *=* 0.03, 95% CI [0.004, 0.05]; RMSEA = 0.02, PCLOSE = .644, 90% CI [0, 0,10]). Each model provided a good fit.

To rule out the alternative temporal order of the mediating variables, we conducted an exploratory path analysis with perceived responsiveness and the halo effects in the reverse order. These results indicate poor fitting models and are presented in the Supplementary Materials.

**Table 2:** Path Models Testing Perceived Expresser Responsiveness and Halo Effects as Mediators Between Expression Type and Affiliative Attitudes and Intentions Toward the Expresser

	b	*SE*	β	95% CI		RMSEA	
				Lower Limit	Upper Limit	Value	95% CI
Trust						0.02	[0, 0.10]
c	0.22^***^	0.05	0.24^***^	0.14	0.33		
d1	0.31^***^	0.07	0.31^***^	0.17	0.45		
d2	0.31^***^	0.07	0.28^***^	0.15	0.41		
MH Indirect Effect	0.11^**^	0.04	0.04^**^	0.02	0.08		
EPH Indirect Effect	0.09^**^	0.03	0.03^**^	0.01	0.06		
Total Effect	0.34^***^	0.08	0.14^***^	0.07	0.20		
Closeness						0.03	[0, 0.10]
c	0.07	0.07	0.07	–0.06	0.19		
d1	0.30^**^	0.11	0.25^**^	0.07	0.43		
d2	0.31^**^	0.11	0.24^**^	0.07	0.40		
MH Indirect Effect	0.11^*^	0.05	0.04^*^	0.01	0.07		
EPH Indirect Effect	0.09^*^	0.04	0.03^*^	0.01	0.06		
Total Effect	0.25^***^	0.08	0.08^***^	0.04	0.13		
Proximity seeking						0.02	[0, 0.10]
c	0.18^***^	0.05	0.18^***^	0.09	0.27		
d1	0.46^***^	0.07	0.42^***^	0.29	0.55		
d2	0.30^**^	0.07	0.26^**^	0.14	0.37		
MH Indirect Effect	0.17^**^	0.05	0.06^**^	0.03	0.10		
EPH Indirect Effect	0.09^**^	0.03	0.03^**^	0.01	0.06		
Total Effect	0.37^***^	0.09	0.14^***^	0.07	0.20		
Willingness to capitalize						0.02	[0, 0.10]
c	0.35^***^	0.06	0.31^***^	0.20	0.41		
d1	0.28^**^	0.10	0.23^**^	0.08	0.39		
d2	0.28^**^	0.10	0.21^**^	0.06	0.35		
MH Indirect Effect	0.10^*^	0.04	0.04^*^	0.01	0.06		
EPH Indirect Effect	0.08^*^	0.04	0.03^*^	0.004	0.05		
Total Effect	0.40^***^	0.09	0.13^***^	0.07	0.19		

Estimates are listed by path label presented in Fig. 3. MH = Moral Halo; EPH = Expanded Positive Halo; RMSEA = root-mean-square error of approximation

^***^p < .001, ^**^p < .01, ^*^p < .05

## Discussion

The findings from this study show that witnesses ascribe greater sets, or halos, of moral and nonmoral positive traits to targets expressing gratitude than simple positivity in a relevant situation. Replicating past findings (Algoe et al., 2020), we also show expressions of gratitude lead to an increased inference of responsiveness from expressers, which in turn underlies affiliative intentions toward them. However, unlike past work, we show part of the influence of perceived responsiveness flows through moral and nonmoral positive trait inferences, thereby identifying an additional mechanism by which gratitude expressions lead to affiliative intentions (cf. Frank, 1988).

These results suggest gratitude provides a unique signal of responsiveness and traits to third-party observers, which lead witnesses to report more willingness to share personal events with gratitude expressers, more interest in becoming acquainted, and more willingness to trust them than general positivity expressers. In short, witnesses view gratitude expressers, due in no small part to gratitude-induced trait inferences, as desirable social partners with whom they feel closer to and are willing to form a connection that can lay the foundation for a trusting relationship.

### Study 2: Trust from Witnessing Gratitude Promotes Cooperation

Given the findings of Study 1, we sought to confirm the ability of gratitude expressions to engender moral and expanded positive halo effects, and to ascertain if these enhanced personal perceptions would lead people to trust gratitude expressers in an incentivized behavioral task containing financial risk. As noted, the expression of a moral emotion like gratitude is thought to build trust by signaling a commitment to acting with others’ interests in mind (Frank, 1988). Inferred moral and general positive traits of the gratitude expresser, particularly those that inform whether someone is trustworthy, could be expected to lead people to attribute a virtuous character and unselfish intentions to the grateful person. Accordingly, we primarily focused on the variable of trust, a principal determinant of cooperation (Axelrod, 1984; Balliet & Van Lange, 2013; Fehr & Rockenbach, 2003; Gambetta, 1988), and assessed whether trust of an unfamiliar gratitude expresser would promote witness cooperation with them in a financial dilemma in which the expresser had to control their self-interest to achieve a shared gain.

## Method

### Participants

We set the target sample size to be 352 based on an effect size that was smaller than those observed in Study 1 and would achieve power = 0.80 at an alpha level = 0.05. As there are several parameters with different effect sizes in the path model, we selected *d* = 0.30 as the minimum effect size of interest as it is below the boundary of smaller effects in the mediation analyses. After accounting for possible loss of 5% data due to data quality issues (e.g., participant technical issues with the videos), we recruited a target sample size of 371. Sensitivity analysis confirmed that we had power = 0.80 to detect the effect size *d* = 0.30.

Participants were adults living in the U.S. recruited through Prolific (Prolific, 2026). They were required to have a 95%+ approval rating and fluency in English. We excluded 14 participants for failing to correctly answer the same attention check question used in Study 1, six participants who had technical issues that prevented them from finishing either or both of the videos in the survey, and six participants who had extreme values for their survey completion time. We followed the same method used in Study 1 for the identification and removal of outliers. The final sample consisted of 345 participants aged 18–80 years (*M* = 38.17, *SD* = 12.70), of whom 209 (61%) were female and 135 (39%) male. One individual self-described their gender. The ethnic composition of the sample was as follows: 56.52% White/Caucasian, 31.88% Black/African American, 4.35% Multiracial, 1.45% Southeast Asian, 1.16% East Asian, and 0.87% South Asian, 0.58% Native American, and 0.58% Middle Eastern. Five participants did not provide their race and four self-described.

### Procedure

Participants completed the same procedure as in Study 1 with minor changes. To focus on the expresser as the target of participants’ perceptions and behavior, some measures from the survey were removed and an economic decision-making game was added. Participants first learned the rules of the economic decision-making game that would later be played at the end of the survey. They watched a video presenting the rules of the game with accompanying visuals and then answered comprehension check questions to ensure they understood how the game is played. Next, participants were randomly assigned to either the Positive Affect Expression Condition (*n* = 169) or Gratitude Expression Condition (*n* = 176) to determine whether they subsequently watched the video depicting either the positivity or gratitude expression. The videos were the same as those used in Study 1. After watching the condition-appropriate video, participants completed questionnaires asking about their perceptions of the expresser’s traits and behavior, their trust of the expresser, and demographic information. Lastly, participants played the economic decision-making game. Participants were fully debriefed at the end of the survey.

### Measures

The measures included in this study captured perceptions of the expresser’s moral traits and general positive traits, the expresser’s observed level of gratitude, perceived expresser responsiveness, and trust of the expresser. These measures were identical to those used in Study 1 with the addition of an economic decision-making game to measure cooperation.

#### Cooperation

To demonstrate their cooperation with the expresser, participants played the “give some dilemma game” (GSDG; DeSteno et al., 2010; Van Lange & Kuhlman, 1994). This game assesses selfish vs. communal behavior by observing whether participants choose to keep their tokens, worth a dollar amount, to maximize their own financial outcome or share their tokens with the other player to benefit both parties. Two players begin the game with four tokens, each worth $1 to them but $2 to the other player, and privately decide how many tokens to allocate to one another. A self-interested decision is best made by giving zero tokens, ensuring one finishes the game with $4, and up to $12 if the other player sends all their tokens. To achieve the most cooperative outcome, both players send all their tokens, resulting in each player finishing the game with $8. Participants in this study were told they would be playing an economic game with Worker 1 from the video who had already made their decision at the time of the video recording and that they would not see Worker 1’s decision. Participants were instructed to, “Please indicate the number of tokens to send to the other player” and responded on a 0–4 numerical scale to designate the number of tokens they wanted to send to Worker 1. Additionally, we told participants we would randomly select 10 players to earn the monetary amount they ended the game with as a bonus payment for their participation in the study. In reality, Worker 1 did not send the participant any tokens, but we did pay winners of the bonus payment the dollar amount of the tokens they kept for themselves ($1 per token) plus the value of two tokens ($4 total), which was the average number of tokens sent in this game.

## Results

### Manipulation Check

An independent samples t-test revealed the manipulation was successful. The expresser in the gratitude video was rated by witnesses as conveying more gratitude (*M* = 5.13, *SD* = 0.90) than the expresser in the positivity video (*M* = 4.79, *SD* = 1.44), *t*(343) = 2.35, *p* = .020, *d* = 0.25, 95% CI [0.04, 0.47].

### Halo Effects as a Function of Emotional Expression

Mirroring the principal finding from Study 1, a 2 (expression type: positivity, gratitude) x 2 (halo type: moral, expanded positive) mixed ANOVA revealed a significant main effect for expression type, *F*(1, 343) = 6.21, *p* = .013, ηp^2^ = 0.02, and a main effect for halo type, *F*(1, 343) = 11.06, *p* < .001, ηp^2^ < 0.03. Once again, the interaction between expression type and halo type was not significant, *F*(1, 343) = 1.86, *p* = .174, ηp^2^ < 0.01. Replicating Study 1 results, witnesses perceived a stronger moral halo for the gratitude expresser (*M* = 4.09, *SD* = 1.13) than for the general positivity expresser (*M* = 3.73, *SD* = 1.28), *t*(343) = 2.75, *p* = .006, *d* = 0.30, 95% CI [0.08, 0.51]. Witnesses also perceived a stronger expanded positive halo when observing a gratitude expression (*M* = 3.92, *SD* = 1.15) than a positivity expression (*M* = 3.66, *SD* = 1.22), *t*(343) = 2.02, *p* = .044, *d* = 0.22, 95% CI [0.005, 0.43].

### Halos as Mediators of Cooperation with Expresser

**Fig. 4:**
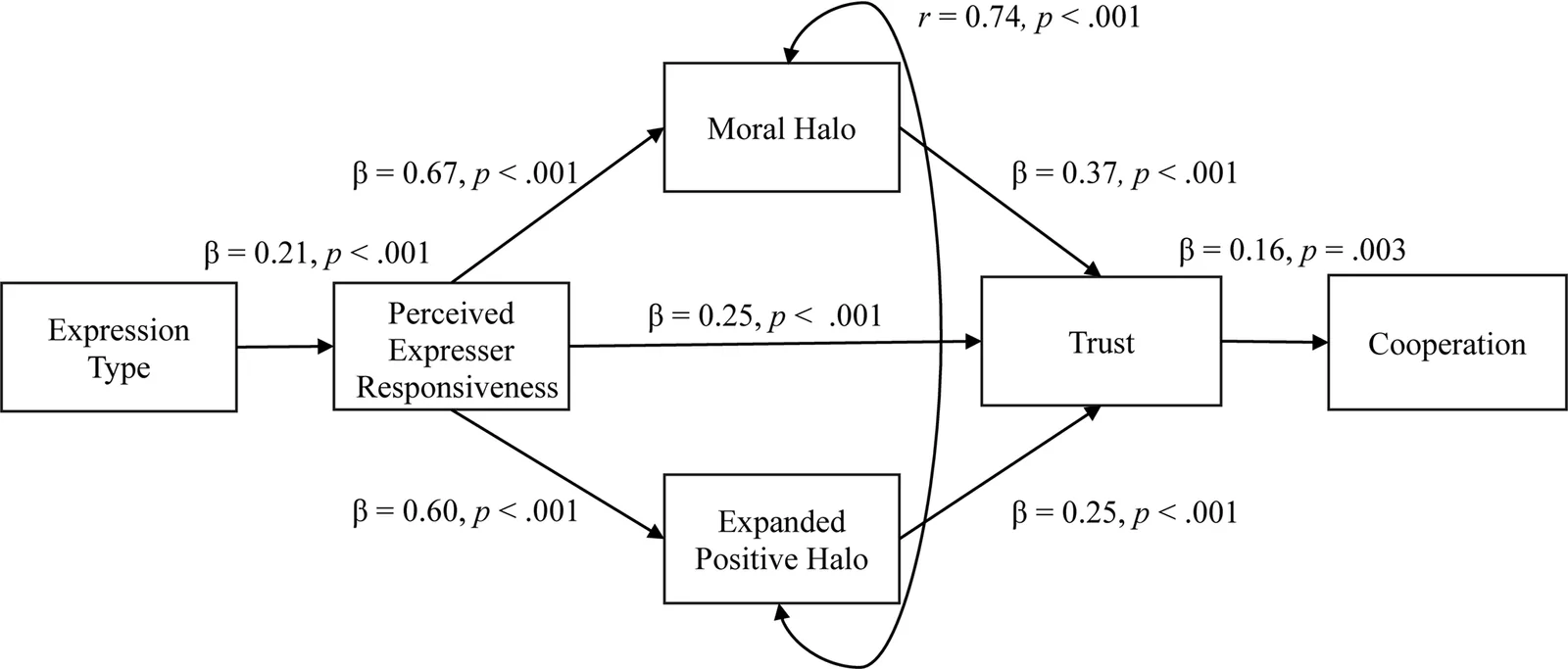
Perceived Expresser Responsiveness, Subsequent Halo Effects, and Trust Mediate Expression Type and Cooperation with the Gratitude Expresser. *Note. *Witnesses perceived greater responsiveness from a gratitude expresser than a positivity expresser, which, in turn, promoted moral and expanded positive halo effects of the gratitude expresser, engendering trust of that expresser and ultimately greater cooperation with them. ^*a*^Expression type was coded as 0 = Positive Affect Expression and 1 = Gratitude Expression. ^*b*^This is a simplified model with the variances of the halo effects correlated. The double-headed curved arrow represents this correlation

Next, we assessed whether witnessing a gratitude expression by a target led to increased cooperative behavior with the expresser via one or both halo effects. To accomplish this, we added cooperation as the final outcome variable to the same path model used in Study 1, but with trust as the primary mediator for cooperation (see Fig. 4). Similarly to Study 1, we removed nonsignificant direct paths from expression type to the halos and, specific to this model, trimmed the nonsignificant paths from the halos to cooperation to achieve good model fit. The results of this path model demonstrated partial mediation with significant indirect effects through both halos.^3^

Analyses showed that the indirect effects from the type of expression witnessed through perceived responsiveness for each halo and through trust to the ultimate financial decision were significant (moral halo indirect effect: *b* = 0.02, *SE* = 0.01, *p* = .031, β *=* 0.01, 95% CI [0.001, 0.02]; expanded positive halo indirect effect: *b* = 0.01, *SE* = 0.01, *p* = .045, β *=* 0.01, 95% CI [0.0001, 0.01]; RMSEA = 0, PCLOSE = .967, 90% CI [0, 0.04]), thereby confirming that witnessing a gratitude expression increases cooperation on a risky task via its effects on person perception. Finally, type of emotional expression was also found to have a significant indirect effect through perceived responsiveness and trust on cooperation (*b* = 0.02, *SE* = 0.01, *p* = .031, β *=* 0.01, 95% CI [0.001, 0.02]), demonstrating partial, as opposed to full, mediation via the halo effects.

## Discussion

Replicating Study 1, the results demonstrate that observers tend to believe those who express gratitude compared to simple positivity are more likely to possesses moral and general positive traits, and these positive impressions, along with a perceived sense of expressers’ increased responsivity, lead them to place more trust in gratitude expressers. In addition, the findings of Study 2 confirm that these gratitude-driven changes in trust directly affect participants’ willingness to share resources under conditions of risk. This finding is of great importance, as it demonstrates the ecological validity of self-reports of a willingness to trust expressers and is among the first to show the ability of gratitude expressions to increase cooperation when true losses are on the line.

### General Discussion

The present studies extend our understanding of how expressions of gratitude affect third-party witnesses’ inferences about and reactions to social targets. Replicating previous work (Algoe et al., 2020), these studies show that observers of gratitude expressions perceive expressers as more socially responsive and as attractive partners with whom to affiliate. They also provide novel evidence for an additional mechanism underlying the links between gratitude expressions and desires to affiliate and cooperate with expressers. In accord with Frank’s (1988) theory of the signaling function of moral emotions, these studies confirm that people ascribe increased moral and general positive traits to gratitude expressers, and these sets of perceived traits separately shape affiliative desires and even cooperative behaviors under conditions of risk. In so doing, these findings complement and integrate a set of differing proposed mechanisms.

Taken together, these findings demonstrate a single instance of observing a previously unknown individual expressing gratitude is taken as sufficient evidence to suggest the expresser possesses a host of moral and general positive traits. Of import, the scope of inferred traits extends beyond those previously measured (e.g., warmth, competence; Algoe et al., 2020; Fehr et al., 2025; Percival & Pulford, 2020; Ritzenhöfer et al., 2017), thereby allowing a fuller examination of the causal role different trait inference categories might play. Thus, gratitude expressions can be understood to function as thin slices—brief observations of another person’s behavior—that are taken to provide enough information for observers to make predictions about a person’s traits and, therefore, confident predictions about their social behaviors (Ambady & Rosenthal, 1992).

By thanking a benefactor, a gratitude expresser signals they believe their benefactor went above and beyond normative relationship expectations and their actions are valued (Yu & Chaudhry, 2024). As a moral emotion communicating this responsive behavior, it follows that an expression of gratitude first and foremost signals an expresser’s capacity to understand, validate, and care for others, which leads to inferences about their stable moral qualities. However, some general positive traits ascribed to the gratitude expresser do not seem inherently linked to responsive behavior but, as noted, are likely accounted for by the density hypothesis (Unkelbach et al., 2008), which suggests other positive traits come to mind easily when making moral trait inferences. As such, they provide an additional route capable of enhancing affiliative intentions and behaviors toward expressers.

A potentially positive consequence of believing unfamiliar gratitude expressers are morally good and bear an array of positive qualities is interest in connecting with them as new social partners (e.g., proximity seeking, willingness to capitalize) and actually leaning into relationship-forming behaviors with them (e.g., cooperation). Across romantic partner and stranger dyads, expressions of gratitude promote high-quality relationships when the gratitude expresser or benefactor is perceived as responsive (e.g., Algoe et al., 2008; Algoe et al., 2010; Algoe et al., 2013; Lambert & Fincham, 2011; Williams & Bartlett, 2015). Research has only recently begun to investigate gratitude’s group-level social function of bringing together third-party witnesses and the individuals in the gratitude interaction to form high-quality relationships (e.g., Algoe et al., 2020). As our research suggests, gratitude expressions can function in an aligned way with strangers—the grateful person’s responsiveness and presumed favorable attributes serve as signals of their potential as a high-quality social partner from the perspective of independent observers. This assessment may lead to observers’ willingness to take initial steps to form cooperative and supportive relationships with a novel partner.

### Limitations and Future Directions

These experiments examined the ways in which observing people express gratitude to an unfamiliar other alters perceptions of them. One potential limitation of this work is the generalizability of the results to situations where witnesses know and have a previous relationship with the individual expressing gratitude. Observations of behaviors from a familiar social partner offer a sliver of information to be weighed against a history of that person’s actions and established opinions of their character. Consequently, we expect strangers’ expressions of gratitude to be seen as stronger signals of information relative to gratitude expressed by a close other.

The video stimuli depicting a gratitude interaction between work colleagues provided a naturalistic context for examining witnessing effects of gratitude. However, in order to maximize experimental control, both the genders of the targets and their relative power/status were held constant, leading to a second potential limitation. It could well be that interactions among these and related variables could significantly moderate the basic effects we identified.

## Conclusion

The results of the present study offer new insight into how gratitude expressions to a helpful individual shape observers’ perceptions of outwardly grateful people and encourage witnesses to form social bonds with them. Expressing gratitude broadcasts information about the expresser’s responsiveness to people outside the gratitude interaction, from which observers form favorable impressions of the gratitude expresser as two positive yet theoretically distinct halo effects. These halo effects, in turn, give rise to intentions and behaviors that are likely to support relationship formation with the gratitude expresser.

## Supplementary Information

Below is the link to the electronic supplementary material.

Supplementary Material 1

## Footnotes

1 The mean survey completion time when the outliers were removed from the sample was 13.24 min, and the mean outlier time was 41.84 min. A reasonable interpretation for this almost tripling of time is participants turned their attention to other tasks or entertainment options while completing the study.

2 Given the high correlation between the two halos, we performed a confirmatory factor analysis to determine whether the two halos were distinguishable. A chi-square difference test showed a two-factor model with separate halos had a better fit than a single-factor model with one all-inclusive halo, Δχ^2^ (1) = 373.38, *p* < .001.

3 A confirmatory factor analysis showed a better model fit for the two-factor model of halos compared to a single-factor model, Δχ^2^ (1) = 329.09, *p* < .001, replicating Study 1 results.

## Additional Information

### Data Availability

Study 1 was pre-registered and is available on the Open Science Framework at (https://osf.io/wngsy/overview?view_only=ba5ff877e0444da6b7bb98ed71b9eb40) . All study materials, data, and code for both Study 1 and Study 2 are available at (https://osf.io/eb4cs/overview?view_only=708e0a29e05e4cc48576c52a6c962dbb).
