# Correction: Gleaning Information from a “Thank You”: How Witnessing Expressions of Gratitude Shape Perceptions of and Actions Toward Others

**DOI:** 10.1007/s42761-026-00397-2

**Published:** 2026-08-05

**Authors:** Alexandra M. Gray, David DeSteno

**Affiliations:** Department of Psychology, Northeastern University, Boston, MA 02115 USA

**Correction to: Affective Science**

**https://doi.org/10.1007/s42761-026-00389-2**

The authors noticed about the formatting of the path figures. Some boxes in the path figures have asymmetrical spacing between the text and perimeters of the box and, overall, the figures are blurry. Most importantly, the last figure is almost unintelligible with the text orientation.

**Figure:**
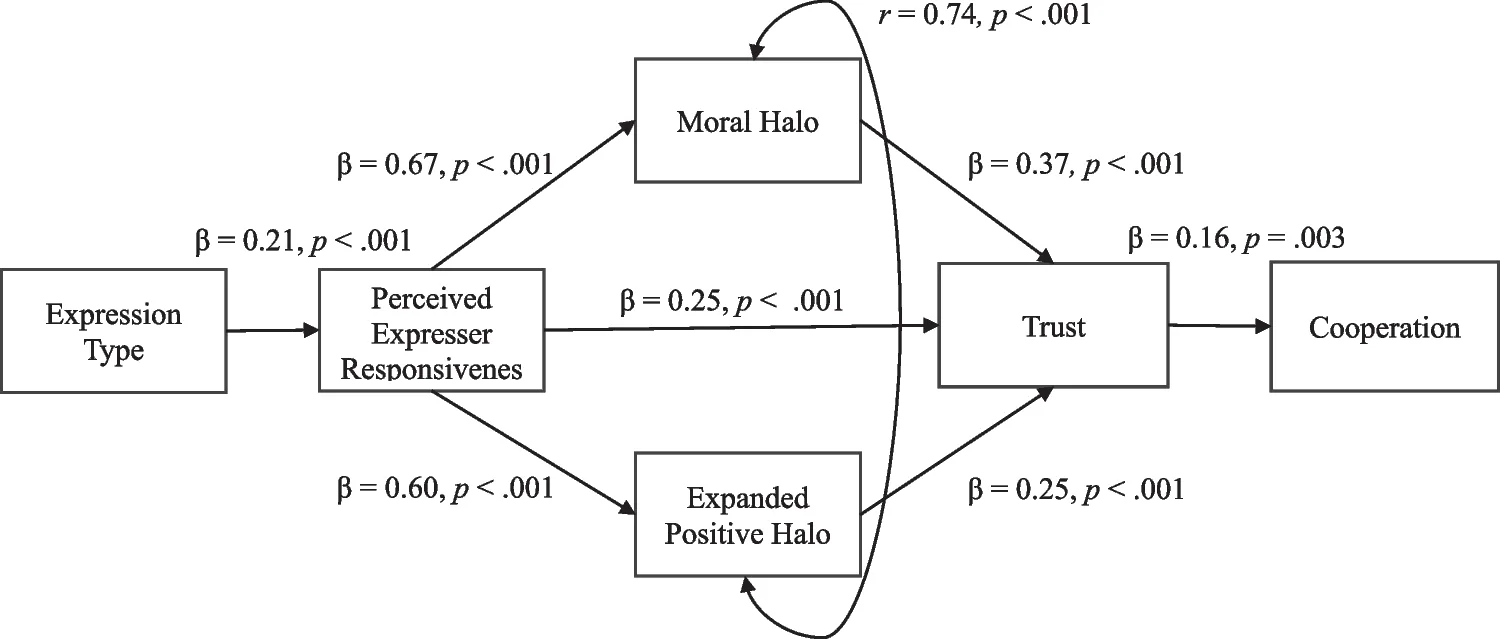

The original article has been corrected.

