# Supplementary material for: Gleaning Information from a “Thank You”: How Witnessing Expressions of Gratitude Shape Perceptions of and Actions Toward Others
Source: Affect Sci. 2026 Jul 15;7(3):420–32. doi: 10.1007/s42761-026-00389-2 (PMC13582724; doi:10.1007/s42761-026-00389-2)
Supplement: Supplementary file 1 [file 42761_2026_389_MOESM1_ESM.odt]

Affective Science
Gleaning Information from a “Thank You”: How Witnessing Expressions of Gratitude Shape Perceptions of and Actions Toward Others 
Alexandra M. Gray, David DeSteno


Correspondence regarding this article should be addressed to Alexandra M. Gray, Department of Psychology, Northeastern University, Boston, MA, 02115, United States.
Email: gray.al@northeastern.edu 
Emotional Expression Norming 
A separate sample was used to standardize the two videos depicting either a gratitude or positivity expression with respect to their overall perceived positivity. Participants (N = 63) in this sample were undergraduates who completed an online survey. They were aged 18-22 years (M = 18.9, SD = 0.96; 58.7% female) and 41.3% were White, 19% East Asian, 9.5% Black/African American, 9.5% South Asian, 7.9% Multiracial, and 1.6% Southeast Asian. Five did not provide their race and one self-described. Participants earned course credit for taking part in the study. 
Participants were randomly assigned to watch a shortened version of either the positivity video (n = 32) or gratitude video (n = 31) that began with the benefactor starting to help resolve the computer issue. To supplement the information removed from the 30-second video clips, text in the survey explained that participants would watch a video of two workers who were creating long client review presentations when one of the computers glitched. Text further described that although the malfunction jeopardized the worker’s progress on their presentation, the computer would be fixed, and they were to rate the workers’ reaction to the solution of their problem. 
Independent samples t-tests revealed there were no differences between the positivity expression and gratitude expression in perceived positive feeling (Mpositivity expression = 3.50, SDpositivity expression = 1.68; Mgratitude expression = 3.97, SDgratitude expression = 1.66, t(61) = 1.11, p = .272, d = 0.28, 95% CI [–0.23, 0.79]), positive expressivity of the worker (Mpositivity expression = 3.47, SDpositivity expression = 1.41; Mgratitude expression = 3.68, SDgratitude expression = 1.42; t(61) = 0.58, p = .562, d = 0.15, 95% CI [–0.36, 0.65]), emotionality of the expression (Mpositivity expression = 3.66, SDpositivity expression = 1.49; Mgratitude expression = 3.61, SDgratitude expression = 1.38, t(61) = 0.12, p = .905, d = 0.03, 95% CI [–0.53, 0.36]), and intensity of the expression (Mpositivity expression = 3.31, SDpositivity expression = 1.33; Mgratitude expression = 3.45, SDgratitude expression = 1.09, t(61) = 0.45, p = .652, d = 0.11, 95% CI [–0.39, 0.62]).


Halos as Mediators of Assumptions of the Expresser’s Social Network and Interpersonal Relations
	Expanding on what witness perceptions result from halo effects on the gratitude expresser, and as a small part of our pre-registered hypotheses, we investigated whether third-party perceptions of halo effects on the gratitude expresser promote assumptions of the expresser’s social network and interpersonal relations. However, emotional expressions may not emit signals strong enough for making judgements about an unknown expresser’s social life. To capture witness predictions about the expresser’s social life, participants were asked whether they believed the expresser has a large social network, strong relationships with others, quality interactions with strangers, and is prosocial.
Results and Conclusion
Using identical structure to the parallel mediation models in the main paper, we assessed whether perceived responsiveness of the expresser, who conveyed either gratitude or positivity, predicted both the moral and expanded positive halos as parallel mediators on each assumption (see Figure 3 for the structure of the model). The association between expression type and witness assumptions about the expresser’s social network and interpersonal relations with others was either not mediated or was partially mediated by perceived responsiveness and subsequent halo effects (see Table S1). Expression type did not exhibit indirect effects through perceived responsiveness and both halos on perceptions of the expresser’s social network size and interaction quality with strangers. However, expression type did yield significant indirect effects through perceived responsiveness and both halos on perceptions of the expresser’s relationship quality with others and prosociality. 
Unlike witnesses’ affiliative attitudes and intentions with the gratitude expresser, perceptions of the gratitude expresser’s responsiveness and halos predicted some assumptions about the expresser’s social network and interpersonal relations with others but did not support the rationale for these speculations in all cases. The findings suggest inferred character across moral and general positive traits are not adequate information for predicting an unknown gratitude expresser’s social network size and interaction quality with strangers. Additional information beyond character is needed to draw such conclusions. However, the gratitude expresser’s ostensible moral and non-moral positive qualities contributed to witnesses believing the gratitude expresser has high-quality relationships with those in their network and helps others in need. That is, the gratitude expresser’s display of responsiveness and assumed traits, from the perspective of the witness, in part speak to their ability to maintain good relationships with close others and put others’ needs before their own. Interestingly, all assumptions about the grateful person’s social life were consistently predicted by the expanded positive halo but not the moral halo. Perceived character consisting of an array of general positive qualities, including intelligence, optimism, warmth, and collaborativeness, may be credited as more important for maintaining a large social circle and quality relationships and interactions than one’s moral goodness. 


Table S1
Path Models Testing Perceived Expresser Responsiveness and Halo Effects as Mediators between Expression Type and Assumptions of the Expresser’s Social Life

b
SE
β
95% CI
RMSEA


Lower limit
Upper Limit
Value
95% CI
Perceived Social Network Size


0.05
[0, 0.12]
c
0.07
0.05
0.08
–0.05
0.20

d1
0.05
0.09
0.06
–0.12
0.23

d2
0.42***
0.08
0.41***
0.25
0.57

MH Indirect Effect
0.02
0.03
0.01
–0.02
0.04

EPH Indirect Effect
0.13**
0.04
0.06**
0.02
0.09

Total Effect
0.18***
0.05
0.08***
0.04
0.12

Perceived Relationship Quality with Others


0.05
[0, 0.12]
c
0.24***
0.05
0.25***
0.14
0.36

d1
0.20*
0.08
0.19*
0.03
0.34

d2
0.34**
0.08
0.30**
0.16
0.44

MH Indirect Effect
0.07*
0.04
0.03*
0.001
0.05

EPH Indirect Effect
0.10**
0.04
0.04**
0.01
0.07

Total Effect
0.32***
0.08
0.13***
0.07
0.18

Perceived Interaction Quality with Strangers


0.02
[0, 0.10]
c
0.27***
0.06
0.26***
0.16
0.37

d1
0.17
0.09
0.15
–0.01
0.31

d2
0.37***
0.09
0.31***
0.16
0.45

MH Indirect Effect
0.06
0.04
0.02
–0.003
0.05

EPH Indirect Effect
0.11**
0.04
0.04**
0.01
0.07

Total Effect
0.33***
0.08
0.12***
0.07
0.18

Perceived Prosociality


0.03
[0, 0.10]
c
0.24***
0.05
0.24***
0.14
0.33

d1
0.30**
0.08
0.27**
0.13
0.41

d2
0.39***
0.08
0.32***
0.20
0.45

MH Indirect Effect
0.11**
0.04
0.04**
0.01
0.07

EPH Indirect Effect
0.12**
0.04
0.04**
0.02
0.07

Total Effect
0.37***
0.09
0.14***
0.07
0.20

Note. Estimates are listed by path label presented in Figure 3. MH = Moral Halo; EPH = Expanded Positive Halo; RMSEA = root-mean-square error of approximation.
*** p < .001, ** p < .01, * p < .05
Exploratory Analysis: Halo Effects on the Benefactor
In addition to the analyses for the main study, we conducted an exploratory analysis on witness perceptions of the moral and expanded positive halo effects on the benefactor. People who provide a benefit for someone perform a virtuous act and subsequently receiving a thank you message could signal additional social information about the benefactor above and beyond their prosocial behavior. Previous work found that third-party witnesses of a gratitude expression view benefactors as morally good people (Algoe et al., 2020). We expanded upon this work by examining whether (1) gratitude expressions uniquely elicit witness perceptions of a moral halo on the benefactor and (2) witnesses perceive an expanded positive halo on the benefactor like they do for the gratitude expresser. 
Results and Conclusions
Similar to witness perceptions of someone conveying an emotional expression, witnesses tend to perceive benefactors who receive a gratitude expression more favorably regardless of the positive trait domain of inferred attributes, F(1, 333) = 4.24, p = .040, p2 = 0.01; they tend to believe benefactors are morally good regardless of the emotion expressed in return for their help, F(1, 333) = 156.94, p < .001, p2 = 0.32; and there is no interaction between halo type and expression type on witness perceptions of the benefactor, F(1, 333) = 0.27, p = .602, p2 < 0.01. As illustrated in Figure S1, witnesses’ perception of the moral halo on the benefactor when witnessing the beneficiary express gratitude (M = 5.31, SD = 0.73) was not significantly different from the moral halo on the benefactor when observing the beneficiary express general positivity (M = 5.15, SD = 0.86), t(333) = 1.83, p = .068, d = 0.20, 95% CI [–0.02, 0.42]. Witnesses, however, did perceive a stronger expanded positive halo on the benefactor who received a gratitude expression (M = 5.05, SD = 0.72) than a benefactor who received a positivity expression (M = 4.87, SD = 0.82), t(333) = 2.16, p = .031, d = 0.24, 95% CI [0.02, 0.45].	
Consistent with prior work (Algoe et al., 2020), we found that witnesses of a gratitude expression perceived a moral halo effect on the benefactor. Notably, this perception of goodness did not differ when compared to moral impressions of a benefactor who received a positivity expression in response to their assistance. For individuals who help others and go out of their way to do the right thing, it seems a gratitude expression does not contribute meaningful information about the benefactor’s moral traits beyond the virtuous behavior itself. In contrast, witnesses perceived a stronger expanded positive halo on benefactors who received a gratitude expression than a positivity expression. Verbally communicating one’s gratitude directs praise toward the benefactor for providing a benefit and publicizes a host of the benefactor’s positive qualities in addition to those demonstrated by their good deed. In doing so, gratitude expressions may offer extra signal value about the benefactor’s general positive qualities.

Fig. S1 Stronger Expanded Positive Halo Effect for Benefactor Receiving the Gratitude Expression. 
Note. Witnesses perceived a stronger expanded positive halo on a benefactor who received a gratitude expression than a benefactor who received a positivity expression (p < .05). There was no difference in moral halo between expression types. 
a Error bars represent 95% confidence intervals.


Exploratory Analysis: Reverse Order Mediation
Because of the difficulties in assessing causal directionality in the absence of manipulating relevant variables (i.e., perceived responsiveness and the two halos) in the parallel mediation model, we conducted a path analysis with these variables in the reverse order to provide increased support for the proposed temporal sequence of mediators. We performed a path analysis testing whether the effects of condition on each affiliative attitude and intention were explained by the two halos serving as mediators for perceived responsiveness, which in turn impacted the affiliative attitudes and intentions. We compared the resulting model fit to that of our proposed model. 
When we reversed the temporal sequence of perceived responsiveness and the halo effects, the model fit becomes unacceptable. Using the model where trust is included as the affiliative attitude and intention as an example, the fit index is poor (RMSEA = 0.333, PCLOSE = 0, 90% CI [0.28, 0.39]), declining from that of a good fit with the original theorized order of mediators (RMSEA = 0.02, PCLOSE = .629, 90% CI [0, 0.10]). Therefore, the data do not support the reverse pattern of sequential mediation between expression type and the affiliative attitudes and intentions, lending support to the originally hypothesized mediator order. Because participants observe the expresser validate the benefactor’s efforts to assist her, the expresser’s responsiveness in making that validation would seem to lead to an assessment of her personal qualities rather than the reverse.
